# 

**Published:** 1884-04

**Authors:** 


					APRIL, 1884
THE
AN JOURNAL
>OF-
Oj
SCIENCE
EDITED BY
GORGA-S, M. D., D. D. S.
THIRD SERIES.
no. 12.
BALTIMORE:
SNOWDEN & COWMAN.
LONDON:
CO., 60 PATERNOSTER ROW.
1883.
PAYABLE IN ADYANCE.
$2.50 PER RNNUM.

				

